# Factors associated with nonattendance at annual diabetes check-up in Ningbo, China: a case-control study

**DOI:** 10.3389/fpubh.2023.1247406

**Published:** 2023-12-14

**Authors:** Ling Li, Kaushik Chattopadhyay, Xueyu Li, Jingjia Yu, Miao Xu, Xueqin Chen, Li Li, Jialin Li

**Affiliations:** ^1^Health Science Center, Ningbo University, Ningbo, China; ^2^Department of Endocrinology and Metabolism, The First Affiliated Hospital of Ningbo University, Ningbo, China; ^3^Lifespan and Population Health, School of Medicine, University of Nottingham, Nottingham, United Kingdom; ^4^Department of Traditional Chinese Medicine, The First Affiliated Hospital of Ningbo University, Ningbo, China

**Keywords:** type 2 diabetes mellitus, chronic disease management, annual checkup, attendance, China

## Abstract

**Background:**

Type 2 diabetes mellitus (T2DM) is a grave issue in China. The annual check-up is recommended in clinical guidelines on T2DM. It plays an important role in monitoring and managing the condition and detecting and managing any comorbidities and T2DM-related complications. However, people with T2DM may miss the annual check-up, and the benefits of this check-up are lost. Therefore, this study aimed to determine the factors associated with nonattendance at the annual T2DM check-up in Ningbo, China.

**Methods:**

A case-control study was conducted using the Ningbo National Metabolic Management Center dataset. Cases were people with T2DM who were alive but did not attend the first annual check-up, scheduled between 1 March 2019 and 28 February 2022 (*n* = 1,549). Controls were people with T2DM who were alive and attended the first annual check-up during the same period (*n* = 1,354). The characteristics of cases and controls were compared using logistic regressions.

**Results:**

The odds of being a female [odds ratio (OR) 1.26, 95% confidence interval (CI) 1.06–1.50], alcohol drinker (1.26, 1.06–1.49), and with glycated hemoglobin A1c (HbA1c) ≥7% (1.67, 1.42–1.97) were higher among case patients than controls. The odds of being a high school graduate (0.77, 0.66–0.89) and on standard treatments in addition to lifestyle modification (oral hypoglycemic drug 0.63, 0.42–0.96; oral hypoglycemic drug and injection therapy 0.48, 0.32–0.73) were lower among case patients than controls.

**Conclusion:**

The factors associated with nonattendance at the annual T2DM check-up in Ningbo, China were female sex, not a high school graduate, alcohol drinker, HbA1c ≥7%, and only on lifestyle modification. The study findings should be used for improving attendance at the annual check-up among people with T2DM in Ningbo.

## Introduction

In China, the prevalence of diabetes is around 11% in adults (1). Current estimates suggest that around 130 million adults in China are living with diabetes (1). The rising prevalence of type 2 diabetes mellitus (T2DM) and its health and socio-economic burden are major concerns in China (2, 3). There is no permanent cure for T2DM, and people with T2DM need continuous care throughout their lifespan (4, 5). Therefore, since 2016, China has been successfully running National Metabolic Management Centers (MMCs) (6). MMC provides care to people from 18 to 75 years old and with a range of metabolic conditions, including T2DM (6). People with T2DM are requested to regularly attend the annual check-up.

The optimal management of T2DM includes the annual check-up to: (i) reinforce essential lifestyle changes, (ii) collect blood and urine samples for assessing biochemical parameters [e.g., glycated hemoglobin A1c (HbA1c), fasting and postprandial blood glucose, lipid profile, estimated glomerular filtration rate (eGFR), urinary albumin to creatinine ratio (UACR)], (iii) measure physiological and anthropometric parameters [e.g., blood pressure, body mass index (BMI), waist circumference], and (iv) examine eyes, feet, and peripheral nerves (7). Thereafter, the blood glucose report is used to monitor if T2DM is under control and if not, medicines are adjusted or changed accordingly (7). Other assessments conducted at the annual check-up help to detect and manage any comorbidities and T2DM-related complications (7). The pathway of care also involves specialist referral for poorly controlled T2DM and comorbidities and T2DM-related complications (7). Therefore, due to the importance of the annual T2DM check-up, it is recommended in major national and international clinical guidelines on T2DM (5, 8). However, there can be a huge gap between what is recommended in a clinical guideline and what happens in real practice (9–11). People with T2DM may miss the annual check-up (9–11) and the benefits of the check-up are lost (11). People with T2DM will gain if they regularly attend the annual check-up and follow the advice provided to them, including, but not limited to, lifestyle (12–14).

Ningbo is one of the most economically developed cities in Eastern China (15). The prevalence of T2DM was around 10% in 2018, and the associated factors were age, family history of diabetes, central obesity, and hypertriglyceridemia (16). There are seven MMCs in Ningbo for providing T2DM care to local people (17). The First Affiliated Hospital of Ningbo University is the main and largest one in Ningbo, running since 1st March 2018 (18). This MMC is one of the two provincial-level MMCs in the Zhejiang Province (19). People can visit any of these seven MMCs, and data are fed into the main one (18).

Globally, limited studies have been conducted on the factors associated with nonattendance at the annual T2DM check-up. For example, a cross-sectional study was conducted in Italy and the United States (11, 20), and a cohort study in Luxembourg (21). These are high-income European and American nations, different from China in many aspects. A related study was conducted in Southwest China, however, it was not exactly on nonattendance at the holistic annual T2DM check-up (22). Southwest China is different from Eastern China in many ways, including socioeconomic and cultural aspects (15). Moreover, the study design was cross-sectional, and in the hierarchy of evidence, cross-sectional studies are ranked below cohort and case–control studies (23). Therefore, this study aimed to determine the factors associated with nonattendance at the annual T2DM check-up in Ningbo, China, using a case–control study design. The findings will help local and national stakeholders to develop, evaluate, and implement solutions for improving attendance at the annual check-up among people with T2DM in Ningbo.

## Methods

### Study design and data source

A case-control study was conducted using the Ningbo MMC dataset, led by The First Affiliated Hospital of Ningbo University.

### Outcome

Cases were people with T2DM who were alive but did not attend the first annual check-up at one of the seven MMCs in Ningbo, scheduled between 1 March 2019 and 28 February 2022 (*n* = 1,549). The annual check-up-related details are provided in the background. Controls were people with T2DM who were alive and attended the first annual check-up at one of the seven MMCs in Ningbo, during the same period (*n* = 1,354).

### Exposures

MMC uses a standardized questionnaire for data collection, and the physiological, anthropometric, and biochemical parameters are measured/analyzed by trained nurse/laboratory staff using the standardized protocol (6). The following characteristics (exposures) were explored in this study, and data came from the first visit before the first annual T2DM check-up:

- Self-reported sociodemographic factors:
  - Age (18–39, 40–59, ≥60 years).
  - Sex (male, female).
  - Education (<high school, ≥high school).
  - Occupation (manual worker, non-manual worker, never worked).
  - Household income (≤30,000, >30,000–100,000, >100,000–300,000, >300,000 yuan/year).
  - Number of people in the house (1, 2, ≥3).
- Self-reported lifestyle factors:
  - Smoking (current status).
  - Alcohol consumption (current status).
  - Physical activity [high, medium, low; assessed using the Chinese version of the International Physical Activity Questionnaire-short (IPAQ)] (24).
- Health conditions and management:
  - BMI normal [<18.5 kg/m^2^), underweight (18.5–23.9 kg/m^2^), overweight (24.0–27.9 kg/m^2^), obesity (≥28 kg/m^2^) (25); body weight and height were measured with light clothes and without shoes in standing position using a calibrated automatic digital weight and height scale (HNH-318, Omron, Japan); weight was measured to the nearest 0.1 kg, height was measured to the nearest 0.5 cm, and BMI was calculated as weight in kg divided by height in m^2^].
  - Self-reported number of comorbidities [0, 1, ≥2; hypertension, hyperlipidemia, hyperuricemia, coronary artery disease, stroke, heart failure, cancer, hyperthyroidism, hypothyroidism, Hashimoto disease, thyroid nodule, non-alcoholic fatty liver disease, viral hepatitis, chronic cholecystitis, gallbladder polyps, gallbladder stones, chronic pancreatitis, chronic gastroenteritis, gastroduodenal ulcer, chronic glomerulonephritis, nephrotic syndrome (except diabetic kidney disease), kidney stone, obstructive sleep apnea-hypopnea syndrome, chronic obstructive pulmonary disease, and emphysema were considered as comorbidities].
  - Self-reported duration of T2DM (≤5, >5–10, >10 years) (26).
  - HbA1c [<7%, ≥7% (5); measured using the high-performance liquid chromatographic (HPLC) method (D-10 Hemoglobin Analyzer, Bio-Rad, United States)].
  - Number of diabetic complications (0, 1, ≥2; diabetic nephropathy, diabetic neuropathy, diabetic retinopathy, diabetic foot, and peripheral arterial disease were considered as diabetic complications).
  - T2DM therapeutic regimen [lifestyle modification alone, lifestyle modification+oral hypoglycemic drug (OHD), lifestyle modification+injection therapy (e.g., insulin, glucagon-like peptide 1 (GLP-1) receptor agonist), lifestyle modification+OHD + injection therapy].

### Ethics statement

The study was approved by the Research Ethics Committee of The First Affiliated Hospital of Ningbo University, China (2019-R057). All the patients gave written informed consent to use the routinely collected data on them for research purposes.

### Statistical analyses

Data analyses were conducted using IBM SPSS Statistics version 26.0 for Windows. We calculated the number and percentage for categorical data. The characteristics of cases and controls were compared using: (i) simple logistic regressions for calculating unadjusted odds ratios (ORs), 95% confidence intervals (CIs), and *p* values and (ii) a multiple logistic regression model, using the backward stepwise regression analysis and all the characteristics were included for calculating adjusted ORs and 95% CIs. Additionally, sensitivity analyses (multiple logistic regression models) were conducted: (i) by excluding missing data (unknown) on characteristics and (ii) by including only those characteristics with a *p* value of ≤0.20 in simple logistic regressions.

## Results

53.4% (1,549/2,903) of people with T2DM did not attend the annual check-up (cases). Table 1 reports the comparison of characteristics of cases and controls using simple logistic regressions. Without adjustment and based on a *p* value of ≤0.05, nonattendance at the annual T2DM check-up was found to be associated with age, education, occupation, household income, number of comorbidities, HbA1c, and T2DM therapeutic regimen.

**Table 1:** Comparison of characteristics of cases and controls using simple logistic regressions.

	Cases (1,549) *n* (%)	Controls (1,354) *n* (%)	Unadjusted OR (95% CI)	*p* value
**Age**				0.022
18–39 years	264 (17.1)	279 (20.6)	1	
40–59 years	860 (55.5)	746 (55.1)	1.22 (1.00–1.48)	
≥60 years	425 (27.4)	329 (24.3)	1.37 (1.09–1.70)	
**Sex**				0.073
Male	972 (62.8)	893 (66.0)	1	
Female	577 (37.2)	461 (34.0)	1.15 (0.99–1.34)	
**Education**				<0.001
Below high school	836 (54.0)	636 (47.0)	1	
High school or above	702 (45.3)	716 (52.9)	0.75 (0.64–0.86)	
Unknown	11 (0.7)	2 (0.1)	4.18 (0.92–18.94)	
**Occupation**				0.017
Manual worker	890 (57.5)	717 (53.0)	1	
Non-manual worker	593 (38.3)	586 (43.3)	0.82 (0.70–0.95)	
Never worked	50 (3.2)	45 (3.3)	0.90 (0.59–1.36)	
Unknown	16 (1.0)	6 (0.4)	2.15 (0.84–5.52)	
**Household income**				0.003
≤30,000 yuan/year	134 (8.7)	93 (6.9)	1	
>30,000–100,000 yuan/year	450 (29.1)	356 (26.3)	0.88 (0.65–1.18)	
>100,000–300,000 yuan/year	675 (43.6)	627 (46.3)	0.75 (0.56–1.00)	
>300,000 yuan/year	211 (13.6)	231 (17.1)	0.63 (0.46–0.88)	
Unknown	79 (5.1)	47 (3.5)	1.17 (0.75–1.83)	
**Number of people in the house**				0.118
1	73 (4.7)	67 (4.9)	1	
2	551 (35.6)	445 (32.9)	1.14 (0.80–1.62)	
≥3	914 (59.0)	839 (62.0)	1.00 (0.71–1.41)	
Unknown	11 (0.7)	3 (0.2)	3.36 (0.90–12.59)	
**Smoking (current status)**				0.214
No	1,028 (66.4)	939 (69.4)	1	
Yes	519 (33.5)	414 (30.6)	1.15 (0.98–1.34)	
Unknown	2 (0.1)	1 (0.1)	1.83 (0.17–20.18)	
**Alcohol consumption (current status)**				0.299
No	844 (54.5)	776 (57.3)	1	
Yes	701 (45.3)	574 (42.4)	1.12 (0.97–1.30)	
Unknown	4 (0.3)	4 (0.3)	0.92 (0.23–3.69)	
**Physical activity**				0.934
High	102 (6.6)	95 (7.0)	1	
Medium	758 (48.9)	653 (48.2)	1.08 (0.80–1.46)	
Low	672 (43.4)	589 (43.5)	1.06 (0.79–1.44)	
Unknown	17 (1.1)	17 (1.3)	0.93 (0.45–1.93)	
**BMI**				0.824
Normal	569 (36.7)	475 (35.1)	1	
Underweight	20 (1.3)	19 (1.4)	0.88 (0.46–1.67)	
Overweight	639 (41.3)	570 (42.1)	0.94 (0.79–1.11)	
Obesity	321 (20.7)	290 (21.4)	0.92 (0.76–1.13)	
**Number of comorbidities**				0.038
0	81 (5.2)	74 (5.5)	1	
1	337 (21.8)	243 (17.9)	1.27 (0.89–1.81)	
≥2	1,131 (73.0)	1,037 (76.6)	0.99 (0.72–1.38)	
**Duration of T2DM**				0.099
≤5 years	604 (39.0)	590 (43.6)	1	
>5–10 years	354 (22.9)	287 (21.2)	1.21 (0.99–1.46)	
>10 years	394 (25.4)	319 (23.6)	1.21 (1.00–1.45)	
Unknown	197 (12.7)	158 (11.7)	1.22 (0.96–1.55)	
**HbA1c**				<0.001
<7%	429 (27.7)	507 (37.4)	1	
≥7%	1,081 (69.8)	805 (59.5)	1.59 (1.36–1.86)	
Unknown	39 (2.5)	42 (3.1)	1.10 (0.70–1.73)	
**Number of diabetic complications**				0.106
0	585 (37.7)	556 (41.0)	1	
1	610 (39.4)	525 (38.8)	1.10 (0.94–1.30)	
≥2	354 (22.9)	273 (20.2)	1.23 (1.01–1.50)	
**T2DM therapeutic regimen**				<0.001
Lifestyle modification alone	75 (4.8)	38 (2.8)	1	
Lifestyle modification+OHD	728 (47.0)	614 (45.3)	0.60 (0.40–0.90)	
Lifestyle modification+injection therapy	77 (5.0)	42 (3.1)	0.93 (0.54–1.60)	
Lifestyle modification+OHD+injection therapy	669 (43.2)	660 (48.7)	0.51 (0.34–0.77)	

OR, odds ratio; CI, confidence interval; BMI, body mass index; T2DM, type 2 diabetes mellitus; HbA1c, glycated hemoglobin A1c; OHD, oral hypoglycemic drug.

Table 2 reports the multiple logistic regression analysis output. The odds of being a female (OR 1.26, 95% CI 1.06–1.50), alcohol drinker (1.26, 1.06–1.49), and with HbA1c ≥7% (1.67, 1.42–1.97) were higher among case patients than controls. The odds of being a high school graduate (0.77, 0.66–0.89) and on standard treatments in addition to lifestyle modification (OHD 0.63, 0.42–0.96; OHD and injection therapy 0.48, 0.32–0.73) were lower among case patients than controls. In the first sensitivity analysis (by excluding missing data), similar results were found except for OHD along with lifestyle modification (not significant). In the second sensitivity analysis (by including those with a *p* value of ≤0.20 in simple regressions), similar results were found except for female sex and alcohol drinkers (not significant).

**Table 2:** Multiple logistic regression analysis output.

	Adjusted OR (95% CI)
**Sex**	
Male	1
Female	1.26 (1.06–1.50)
**Education**	
Below high school	1
High school or above	0.77 (0.66–0.89)
Unknown	4.87 (1.06–22.31)
**Alcohol consumption (current status)**	
No	1
Yes	1.26 (1.06–1.49)
Unknown	0.65 (0.15–2.85)
**Number of comorbidities**	
0	1
1	1.27 (0.88–1.82)
≥2	0.99 (0.71–1.39)
**HbA1c**	
<7%	1
≥7%	1.67 (1.42–1.97)
Unknown	1.09 (0.69–1.74)
**T2DM therapeutic regimen**	
Lifestyle modification alone	1
Lifestyle modification+OHD	0.63 (0.42–0.96)
Lifestyle modification+injection therapy	0.89 (0.51–1.53)
Lifestyle modification+OHD+injection therapy	0.48 (0.32–0.73)

OR, odds ratio; CI, confidence interval; HbA1c, glycated hemoglobin A1c; T2DM, type 2 diabetes mellitus; OHD, oral hypoglycemic drug.

## Discussion

This study determined the factors associated with nonattendance at the annual T2DM check-up in Ningbo, China, and 53% of patients did not attend (cases). The odds of being a female were higher among case patients than controls. A qualitative study should be conducted among them to explore the reasons behind such an action. The study conducted in Luxembourg reported a higher nonattendance at the annual T2DM check-up among males (21). This opposite finding could be unique in our study population and setting. However, no association between sex and nonattendance at the annual T2DM check-up was found in the study conducted in the United States (11), similar to our sensitivity analysis. Nevertheless, this needs attention as women with T2DM compared to men with T2DM have a higher risk of developing coronary heart disease (27). As expected, we found that the odds of being a high school graduate were lower among case patients than controls. Prior formal education can play a key role in T2DM management-related health education and promotion, including access to healthcare and uptake and adherence to medical advice and therapies (28–31). We found that the odds of being an alcohol drinker were higher among case patients than controls, but no association was found in the sensitivity analysis. Nonetheless, missing the annual check-up could be due to the well-known adverse effects of alcohol on health such as learning and memory problems including dementia (32). Studies conducted on self-management in T2DM and healthcare utilization also reported the negative impact of alcohol (33–35).

In our study, the odds of having HbA1c ≥7% were higher among case patients than controls. The finding is consistent with studies conducted on nonattendance at follow-up visits among people with T2DM (36, 37). It should be noted that in T2DM, hyperglycemia is one of the most important causes of vascular complications and even premature death (38–40). We found that the odds of being on standard treatments in addition to lifestyle modification were lower among case patients than controls. The finding is consistent with a study conducted on nonattendance at follow-up visits among people with T2DM (41). However, in our sensitivity analysis, the association between lifestyle modification and OHD treatment and nonattendance at the annual T2DM check-up was not significant. Nonetheless, the prescription of T2DM medicine by a clinician can act as a facilitator in T2DM management (31). This could be the reason for attending the annual check-up by people with T2DM. On the opposite, those only on lifestyle modification, an initial step in T2DM management, might have taken the disease lightly or have been expecting more than behavior change advice. Again, a qualitative study should be able to shed light on the reasons behind such an action.

Our study has several strengths and weaknesses. To the best of our knowledge, this was the first study that explored the factors associated with nonattendance at the annual T2DM check-up in Eastern China. We used previously collected routine data on the outcome and exposures, which has its own merits in a case–control study. However, there could be some data quality issues, especially recall bias in self-reported data, e.g., physical activity, number of comorbidities, and duration of T2DM. Although MMC collects data on several comorbidities, however, the list is limited. The findings could be due to other variables which were not present in the dataset and were not adjusted for in multiple logistic regression models, e.g., proximity to a healthcare service provider and availability of health insurance (31, 42). Therefore, future observational studies should measure exposures objectively and include other variables that were missing in our study. Individual matching was not performed. Considering the issue of random error, it is recommended to choose all available controls, instead of choosing matched controls from the available controls, especially when a large number of controls are available (43). Having said this, we conducted multiple logistic regression analyses to adjust for potential confounders. In our study, missing data were generally low (see Table 1 for unknown). Missing data on characteristics were included in the main multiple logistic regression model. There could be some genuine and unique reasons behind nonattendance at the annual T2DM check-up, e.g., migration to another location or fear of COVID-19 associated with in-person visits. Therefore, the above-mentioned qualitative study needs to be conducted to explore the reasons behind such an action. However, compared to the rest of the world, Ningbo consistently had a low number of COVID-19 cases, even during the early stage of the outbreak (44). The lockdown period affecting healthcare services accessibility was from late January 2020 to mid-April 2020, i.e., less than 3 months. During this short lockdown period, alternatives like online consultations were available to people with T2DM, mainly to control their blood glucose levels. In other words, COVID-19 had a limited impact on this study (45). Patients receive the appointment details through a text message 1 month before the annual T2DM check-up. One week before the appointment, patients are reminded through a phone call by a nurse. However, the effectiveness of this strategy in MMCs has not yet been evaluated. Systematic reviews suggest that this strategy is an effective way to improve clinic attendance in general (46–48). The other effective intervention could be patient education (49).

## Conclusion

The factors associated with nonattendance at the annual T2DM check-up in Ningbo, China were female sex, not a high school graduate, alcohol drinker, HbA1c ≥7%, and only on lifestyle modification. The study findings should be used for improving attendance at the annual check-up among people with T2DM in Ningbo.

## Data availability statement

The original contributions presented in the study are included in the article/supplementary material, further inquiries can be directed to the corresponding author.

## Ethics statement

The study was approved by the Research Ethics Committee of The First Affiliated Hospital of Ningbo University, China (2019-R057). All the patients gave written informed consent to use the routinely collected data on them for research purposes.

## Author contributions

JL and KC designed the study. XL cleaned the dataset. LingL and KC analyzed the data and wrote the first draft of the manuscript. LingL, KC, XL, JY, MX, XC, LiL, and JL revised it critically for important intellectual content and approved the final version. All authors contributed to the article and approved the submitted version.
